# Prefrontal dysfunction in pediatric Tourette’s disorder as measured by near-infrared spectroscopy

**DOI:** 10.1186/s12888-015-0472-3

**Published:** 2015-05-03

**Authors:** Kazuhiko Yamamuro, Toyosaku Ota, Junzo Iida, Yoko Nakanishi, Mitsuhiro Uratani, Hiroki Matsuura, Naoko Kishimoto, Shohei Tanaka, Hideki Negoro, Toshifumi Kishimoto

**Affiliations:** Department of Psychiatry, Nara Medical University School of Medicine, 840 Shijyou-cho, Kashihara, Nara, 634-8522 Japan; Faculty of Nursing, Nara Medical University School of Medicine, Kashihara, Nara, Japan; Department of Psychiatry, Nara Prefectural General Rehabilitation Center, Shiki, Nara, Japan; Department of Psychiatry, Tenri Hospital, Tenri, Nara, Japan; Department of Education, Nara University of Education, Nara, Japan

**Keywords:** Near-infrared spectroscopy, Tourette’s disorder, The Stroop Color-Word Task, Tics, Dorsolateral prefrontal cortex

## Abstract

**Background:**

Tourette’s disorder (TD) is a chronic childhood-onset disorder characterized by the presence of multiple motor and vocal tics. Despite strong evidence that the pathophysiology of TD involves structural and functional disturbances of the basal ganglia and cortical frontal areas, in vivo imaging studies have produced conflicting results. Recent developments in near-infrared spectroscopy (NIRS) technology have enabled noninvasive assessment of brain function in people with psychiatric disorders.

**Methods:**

We asked 10 individuals with pediatric TD and 10 healthy controls who were age- and sex- matched to perform the Stroop color-word task during NIRS. We used prefrontal probes and a 24-channel NIRS machine to measure the relative concentrations of oxyhemoglobin (oxy-Hb) every 0.1 s during the task.

**Results:**

We found that oxy-Hb changes in the prefrontal cortex were significantly smaller in the TD group compared with the control group, especially in the left dorsolateral prefrontal cortex.

**Conclusions:**

Our data suggest that individuals with pediatric TD have a reduced prefrontal hemodynamic response as measured by NIRS.

## Background

Tourette’s disorder (TD) is a childhood neuropsychiatric disorder characterized by persistent motor and vocal tics. The prevalence of TD is between 0.05% and 3%, and it is often comorbid with obsessive-compulsive disorder (OCD), attention deficit/hyperactivity disorder (ADHD) and other social and behavioral disturbances (50% of individuals with TD also have ADHD, while 20–60% also have OCD) [1,2]. TD without any comorbidity occurs in only about 10% of patients [3,4].

Several biological hypotheses exist regarding the underlying mechanisms of TD. As these generally center around abnormal dopaminergic function, typical antipsychotics, such as haloperidol and pimozide, are often prescribed to control tic symptoms [5]. Recent improvements in the efficacy and side effect profiles of atypical antipsychotics (relative to classical antipsychotics) have led to an increase in the clinical use of these drugs. Of the available antipsychotics, risperidone is most commonly recommended by experts [6,7]. Paliperidone extended-release, which chemically is a major active metabolite of risperidone (9-hydroxyrisperidone), has been suggested as an efficacious therapy with few side effects in child and adolescent patients with TD [8].

TD is thought to involve the frontal cortex and its connections to subcortical regions, such as the basal ganglia, through frontal cortico-striatal-thalamo-cortical (CSTC) circuits [2,9,10]. Electrophysiological and histological evidence for reduced GABA-ergic tone in these cortical and subcortical brain regions is indicative of reduced local inhibition of neural activity in these motor circuits [11,12]. Specifically, children with TD exhibit reduced grey matter volumes in the basal ganglia [13] and increased grey matter volumes in the dorsolateral prefrontal cortex (DLPFC) [14]. This change in grey matter volume may be related to the continual need to suppress tics, leading to compensatory neural plasticity within the prefrontal cortices. In a diffusion tensor imaging study by Xi et al. [15], the researchers found that children with TD exhibited increased parallel and mean diffusivity in the bilateral putamen, increased perpendicular diffusivity in the right thalamus, reduced anisotropy in the bilateral thalamus, decreased fractional anisotropy in the left globus pallidus, and an increased diffusion coefficient in the bilateral caudate nucleus. Studies of TD patients using single photon emission computed tomography (SPECT) have indicated that these individuals have increased activity in the right frontal lobe [16] and reduced activity in the left caudate and anterior cingulate gyrus compared with controls [17]. In addition, studies of TD patients using positron emission tomography (PET) have reported localized decreases in functional activity in the basal ganglia and thalamus and increased functional activity in the lateral premotor and supplementary motor association cortices, as well as in the midbrain [18]. Consistent with anatomical and imaging findings, functional magnetic resonance imaging (fMRI) studies of TD patients have pointed to abnormalities in frontostriatal function [13,19]. The above-mentioned studies, which used varying methodologies, all reported that individuals with TD exhibit atypical activity in the prefrontal cortex. Thus, it is possible that TD patients have an abnormal prefrontal hemodynamic response.

Multi-channel near- infrared spectroscopy (NIRS) enables the noninvasive detection of neural activity near the surface of the brain using near-infrared light [20,21]. NIRS measures alterations in oxygenated hemoglobin ([oxy-Hb]) and deoxygenated hemoglobin ([deoxy-Hb]) concentrations in micro-blood vessels on the brain surface. Local increases in [oxy-Hb] and decreases in [deoxy-Hb] are indicators of cortical activity [21,22]. Furthermore, changes in [oxy-Hb] have been associated with changes in regional cerebral blood volume, using a combination of NIRS and PET measurements [23,24]. NIRS is a neuroimaging modality that is especially suitable for psychiatric patients for the following reasons [25]. First, because NIRS is relatively insensitive to motion artifact, it can be used in experimental scenarios in which motion may occur, such as while assessing participants who are prone to vocalization. Second, participants can be examined in a natural sitting position, without any surrounding distractions. Third, the cost is much lower than that of other neuroimaging modalities and the setup is very easy. Fourth, the high temporal resolution of NIRS is useful in characterizing the time course of prefrontal activity in people with psychiatric disorders [26,27]. Fifth, Functional studies of pediatric patients using SPECT and PET are rare due to restrictions regarding the use of radioactive materials in young individuals. Accordingly, NIRS has been used to assess brain function in people with many types of psychiatric disorders, including schizophrenia, bipolar disorder, post traumatic disorder, OCD, and ADHD [25-30].

In this study, we used NIRS to investigate the prefrontal hemodynamic response to the Stroop colour-word task in pediatric TD patients. Based on the findings of previous studies that used other neuroimaging techniques to find abnormal activity in the prefrontal cortex in patients with TD, we hypothesized that pediatric TD patients would have an increased prefrontal hemodynamic response, as measured by NIRS. To test this, we used a multi-channel NIRS method to examine changes in prefrontal cerebral blood volume during the Stroop color-word task in children with TD and age- and sex- matched controls.

## Methods

### Participants

Ten TD patients (10 males; mean age 9.20 years ± 2.25 years SD) were recruited from the outpatient clinic of the Department of Psychiatry at Nara Medical University, Japan. All patients were diagnosed with TD according to the DSM-IV-TR. A control group comprising 10 age- and sex- matched healthy individuals (10 males; mean age 9.50 years ± 2.12 years SD) was recruited via local print advertising (Table 1). All participants were right-handed and of Japanese descent. All caregivers of the participants provided written informed consent, and the participants provided verbal assent regarding their participation in the study. This study was approved by the Institutional Review Board at the Nara Medical University.

**Table 1:** **Participant characteristics**

	**TD**	**Control**	**p-value**
	**mean (SD)**	**mean (SD)**	
Number [sex ratio: M:F]	10 [10:0]	10 [10:0]	1.00
Age (years)	9.20 (2.25)	9.50 (2.12)	0.76
Age of onset (years)	7.40 (1.84)		
Duration of illness (months)	20.50 (8.24)		
FIQ (WISC-III)	99.80 (15.32)	97.30 (9.90)	0.67
YGTSS	18.00 (6.75)		
SCWC-1	26.80 (11.14)	42.70 (9.78)	0.003
SCWC-2	26.10 (10.21)	40.20 (8.77)	0.004
SCWC-3	27.20 (12.74)	37.70 (8.11)	0.027

TD, Tourette’s disorder; M, male; F, female; FIQ (WISC-III), Full scale IQ score on the Wechsler Intelligence Scale for Children-Third Edition; YGTSS, Yale Global Tic Severity Scale; SCWC-1, ‘Stroop color-word task number of correct answers first time’; SCWC-2, ‘Stroop color-word task number of correct answers second time’; SCWC-3, ‘Stroop color-word task number of correct answers third time’.

Patients were deemed eligible if they had received a DSM-IV-TR diagnosis of TD, as described in the Kiddie Schedule for Affective Disorders and Schizophrenia for School-Age Children-Present and Lifetime version (K-SADS-PL) [31], and a medical history evaluation. Of the individuals with TD, none had experienced a comorbid major depressive disorder, schizophrenia, OCD, other anxiety disorders, or epilepsy. Five participants had comorbid ADHD (but had not received any previous medication for ADHD).

Exclusion criteria included any neurological disorder, a head injury, a serious medical condition, or a history of substance abuse/dependence. Intellectual level was assessed using the Wechsler Intelligence Scale for Children–Third Edition, and participants with full-scale IQ (FIQ) scores below 70 were also excluded. Seven of the 10 TD patients selected for the study were not medicated for the disorder, whereas the remaining three participants were receiving medication for TD symptoms (two, haloperidol; one, risperidone).

### Assessment of TD symptoms

The Yale Global Tic Severity Scale (YGTSS) [32] is a semi-structured clinical interview designed to assess current tic severity. This scale yields three summary scores: total motor (0–25), total phonic (0–25), and total tic (sum of motor and phonic) scores. The YGTSS also contains an impairment scale (0–50), which evaluates the global level of functional impairment arising from tics.

A higher YGTSS score is associated with higher tic symptom severity.

### The stroop color-word task

We combined the traditional Stroop task with the word reading task, incongruent color naming task, and the color naming task. However, we reconstructed the Stroop task according to previously described methods [33]. The Stroop color-word task consisted of two pages. The items on the first page included the color words RED, GREEN, and BLUE printed in black ink. The items on the second page included the words RED, GREEN, and BLUE printed in red, green, or blue ink, with the limitation that the word meaning and ink color could not match. The items on both pages were randomly distributed, except that no item within a column could follow itself.

Before administering the task, the examiners instructed the participants as follows: “This is to test how quickly you can read aloud the words on the first page, and say the colors of the words on the second page. After you have read the words on the first page for 45 s, we will turn the page immediately. Then you will say the colors of the words on the second page for 45 s. We will repeat this process three times.”

The entire Stroop color-word task sequence consisted of three cycles of 45 s spent reading the first page and then 45 s spent reading the second page (the color-word task). The task ended with 45 s spent reading the first page again, which we designated as the baseline task. We counted the number of correct answers in each cycle, and gave them the following designations: ‘Stroop Color-Word task number of correct answers first time’ (SCWC-1), ‘…second time’ (SCWC-2), and ‘…third time’ (SCWC-3).

We chose to use a stroop color-word task simply because activity in the inferior frontal gyrus is strongly related to Stroop interference [34], and the same task was used in a previous NIRS study of individuals with pediatric OCD and ADHD [28,30]. Thus these methods enabled us to directly compare our results with those of previous studies.

### NIRS measurements

Increased oxy-Hb and decreased deoxy-Hb, as measured by NIRS, have been shown to reflect cortical activation. In animal studies, oxy-Hb has been found to be a highly sensitive indicator of regional cerebral blood flow because the direction of change in deoxy-Hb is determined by the degree of change in venous blood oxygenation and volume [35]. Therefore, we decided to focus on changes in oxy-Hb. We measured oxy-Hb using a 24-channel NIRS machine (Hitachi ETG-4000, Hitachi Medical Corporation, Tokyo, Japan). We measured the absorption of two wavelengths of near-infrared light (760 and 840 nm). Oxy-Hb was calculated as previously described [36]. The inter-probe intervals of the machine were 3.0 cm, and previous reports have established that the machine measures at a point 2–3 cm beneath the scalp, i.e., the surface of the cerebral cortex [37,38].

**Figure 1:**
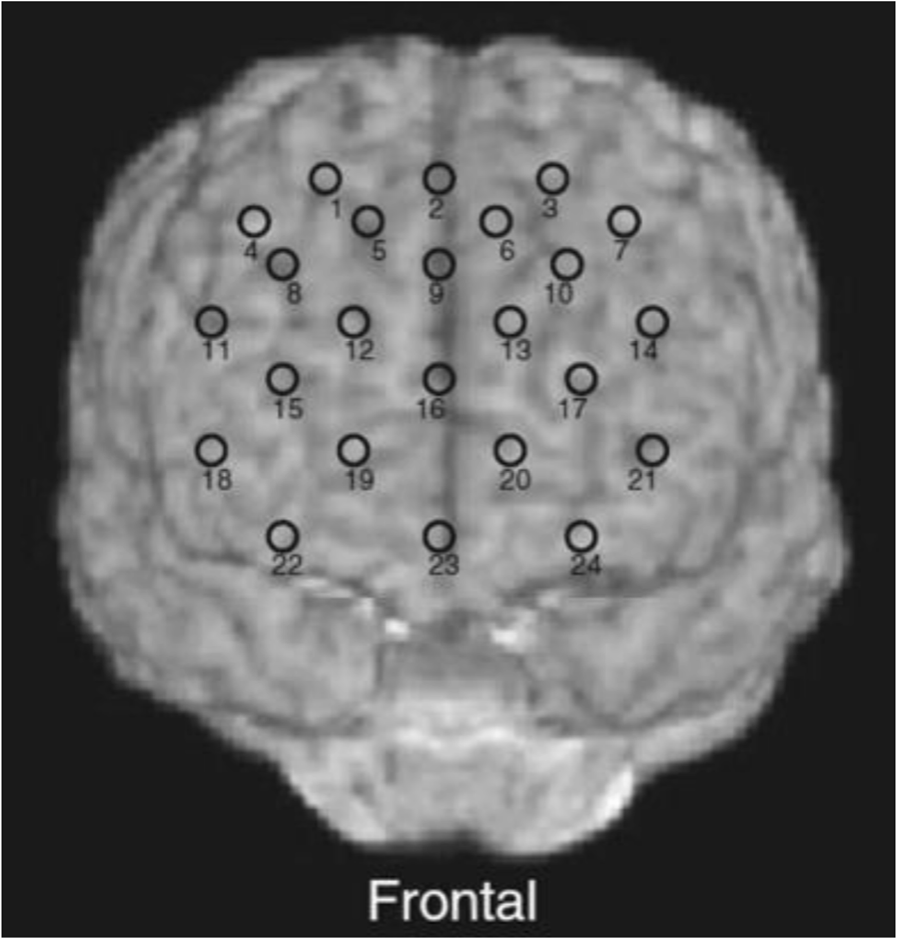
Cortical projection of near-infrared spectroscopy (NIRS) measurement points. The points were mapped onto the anatomical frontal lobes using MRIcro software (MRIcro: developed by Dr. Chris Rorden, available at http://www.mricro.com). Numbers denote the channel numbers for the points of measurement.

The participants were asked adopt a natural sitting position for NIRS measurement. The NIRS probes were placed on the scalp over the prefrontal brain regions. The probes were arranged to measure relative changes in Hb concentration at 24 measurement points comprising an 8 × 8 cm square. The lowest probes were positioned along the Fp1-Fp2 line according to the international 10/20 system commonly used in electroencephalography. Correspondences between the probe positions and the measurement points in the cerebral cortex were confirmed by superimposing the probe positions onto a three-dimensionally reconstructed cerebral cortex of a representative participant in the control group, obtained via MRI (Figure 1). The absorption of near-infrared light was measured with a time resolution of 0.1 s. The data were analyzed using the “integral mode”: the pre-task baseline was calculated as the mean across the 10 s just before the task period, the post-task baseline was calculated as the mean across the 25 s immediately after the task period, and we conducted linear fitting on the data between the two baselines. We used moving average methods to exclude short-term motion artifacts in the analyzed data (moving average window, 5 s).

We attempted to manually exclude motion artifacts by closely monitoring artifact-evoking body movements, such as neck movements, biting, and blinking (identified as being the most influential in a preliminary artifact-evoking study), and by instructing the participants to avoid these movements during the NIRS measurements. Examiners were blind to the treatment condition of the participants.

### Statistical analyses

**Figure 2:**
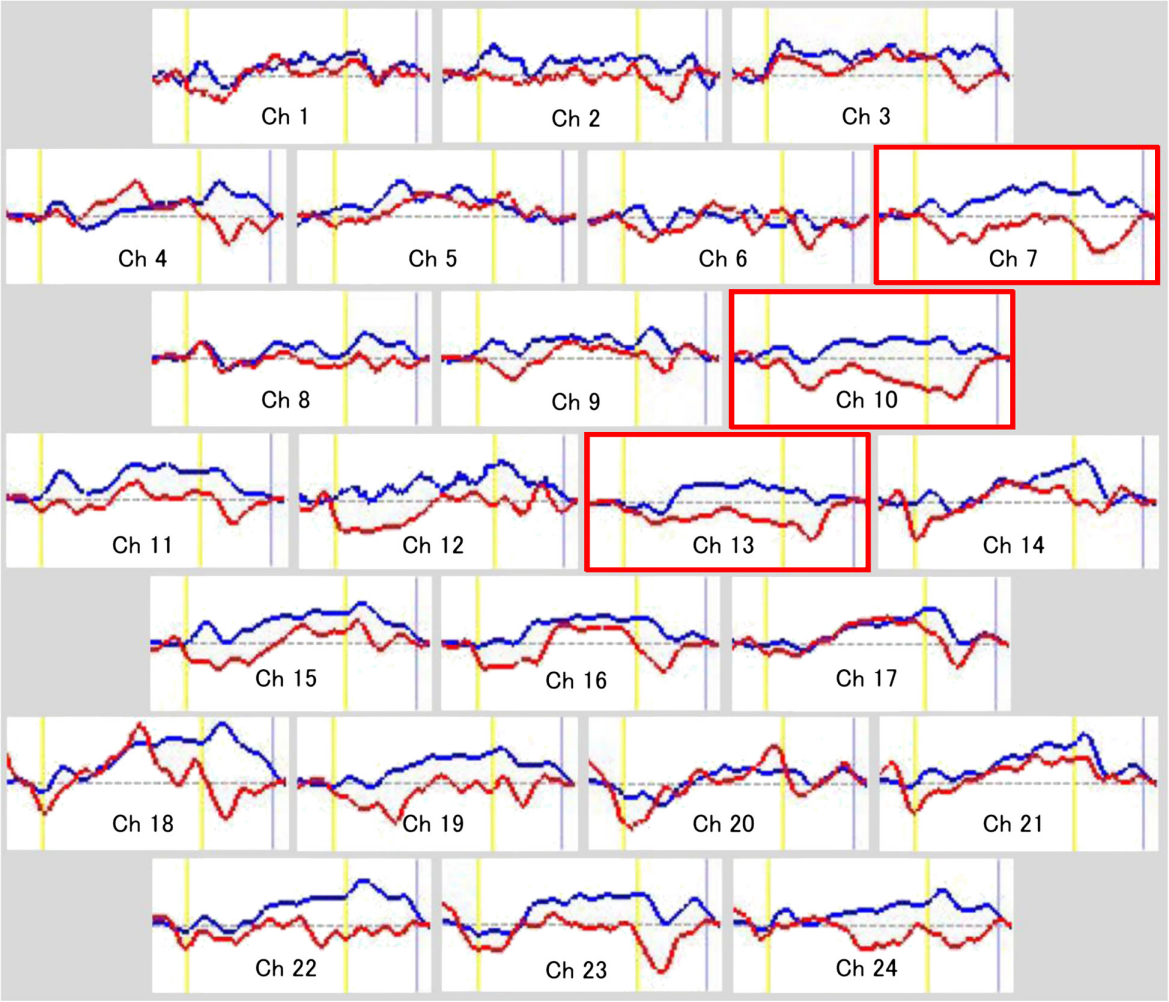
Grand average waveforms showing changes in oxyhemoglobin (oxy-Hb) during the Stroop color-word task in both groups (red lines denote the Tourette’s group and blue lines denote the control group). The task occurred in the interval represented by the yellow lines (the first line indicates the beginning of the task, the second line indicates the end of the task).

We used Student’s *t*-tests to compare Oxy-Hb changes between the two groups by calculating the grand average waveforms every 0.1 s in each channel. This analysis enabled a more detailed comparison of oxy-Hb changes along the time course of the task. Data analyses were conducted using MATLAB 6.5.2 (Mathworks, Natick, MA, USA) and Topo Signal Processing type-G version 2.05 (Hitachi Medical Corporation, Tokyo, Japan). We used OT-A4 version 1.63 K (Hitachi Medical Corporation, Tokyo, Japan) to create the overlap display of the grand average waveforms for both groups in Figure 2 and to calculate the mean oxy-Hb measurements in Table 2. Since we performed 24 paired *t* tests, we conducted the correction for multiple comparisons using the false discovery rate (FDR) (two-tailed; we set the value of *q* specifying the maximum FDR to 0.05, so that on average, there would be no more than 5% false positives [39]). We used PASW Statistics 18.0 J for Windows (SPSS, Tokyo, Japan) for statistical analysis.

**Table 2:** **Mean difference in oxyhemoglobin (oxy-Hb) measurements between the task and post-task periods in 24 channels**

	**TD (mMmm)**		**Control (mMmm)**		**Student’s** ***t*** **test**	**FDR correction**
	**mean**	**SE**	**mean**	**SE**		
Ch 1	0.0024	0.0214	0.0199	0.0148	NS	NS
Ch 2	−0.0078	0.0157	0.0290	0.0216	NS	NS
Ch 3	0.0211	0.0123	0.0485	0.0147	NS	NS
Ch 4	0.0093	0.0220	0.0228	0.0132	NS	NS
Ch 5	0.0186	01.149	0.0308	0.0133	NS	NS
Ch 6	−0.0136	0.0140	−0.0016	0.0095	NS	NS
Ch 7	−0.0283	0.0145	0.0393	0.0137	**	***
Ch 8	−0.0054	0.0211	0.0190	0.0117	NS	NS
Ch 9	0.0044	0.0169	0.0342	0.0111	NS	NS
Ch 10	−0.0035	0.0210	0.0454	0.0079	**	***
Ch 11	−0.0035	0.0210	0.0454	0.0079	*	NS
Ch 12	−0.0202	0.0368	0.0387	0.0126	NS	NS
Ch 13	−0.0332	0.0116	0.0188	0.0099	**	***
Ch 14	0.0039	0.0231	0.0319	0.0051	NS	NS
Ch 15	−0.0019	0.0228	0.0470	0.0090	†	NS
Ch 16	−0.0083	0.0213	0.0340	0.0055	†	NS
Ch 17	0.0151	0.0138	0.0283	0.0056	NS	NS
Ch 18	0.0131	0.0197	0.0651	0.0146	*	NS
Ch 19	−0.0208	0.0281	0.0379	0.0144	†	NS
Ch 20	0.0018	0.0320	0.0089	0.0127	NS	NS
Ch 21	0.0183	0.0301	0.0458	0.0103	NS	NS
Ch 22	−0.0134	0.0202	0.0426	0.0167	*	NS
Ch 23	−0.0219	0.0264	0.0348	0.0165	†	NS
Ch 24	−0.164	0.0227	0.0341	0.0112	†	NS

^†^P < 0.1; *P < 0.05; **P < 0.01; ***P < FDR-corrected P.

Group differences tested using *t*-tests with false discovery rate (FDR) correction.

## Results

### Demographic data

Age, sex, and FIQ did not differ significantly among the TD patients and healthy controls (Table 1). The mean YGTSS score of TD patients was 18.00 (SD, 6.75). We found significant differences in the SCWC-1, SCEC-2, and SCWC-3 scores between the groups.

### Correlation between Stroop task performance and participant characteristics

Because the TD patient and control groups varied considerably in terms of SCWC scores, we calculated Spearman’s ρ correlations for the SCWC scores, age, FIQ, and YGTSS scores (Table 3). In the TD patient group, we found a positive correlation between SCW-1 scores and age (Spearman’s r = 0.693, P < 0.05), and no correlations between the SCWC, FIQ, and YGTSS scores. In the control group, we found a positive correlation between the SCW-1 scores and age (Spearman’s r = 0.628, P < 0.05), and no correlations between the SCWC and FIQ scores.

**Table 3:** **Correlations between Stroop task performance and participant characteristics**

	**TD**			**Control**		
	**SCWC-1**	**SCWC-2**	**SCWC-3**	**SCWC-1**	**SCWC-2**	**SCWC-3**
Age	0.693*	0.618†	0.394	0.628*	0.514	0.584†
FIQ (WISC-III)	0.335	0.302	0.257	−0.353	−0.175	−0.287
YGTSS	0.229	0.229	0.548			

✝*P* < 0.1; *P < 0.05

Tested using Spearman’s correlation test.

TD, Tourette’s disorder; FIQ (WISC-III), Full scale IQ score on the Wechsler Intelligence Scale for Children-Third Edition; YGTSS, Yale Global Tic Severity Scale; SCWC-1, ‘Stroop color-word task number of correct answers first time’; SCWC-2, ‘Stroop color-word task number of correct answers second time’; SCWC-3, ‘Stroop color-word task number of correct answers third time’.

### NIRS data from participants while performing the Stroop color-word task

**Figure 3:**
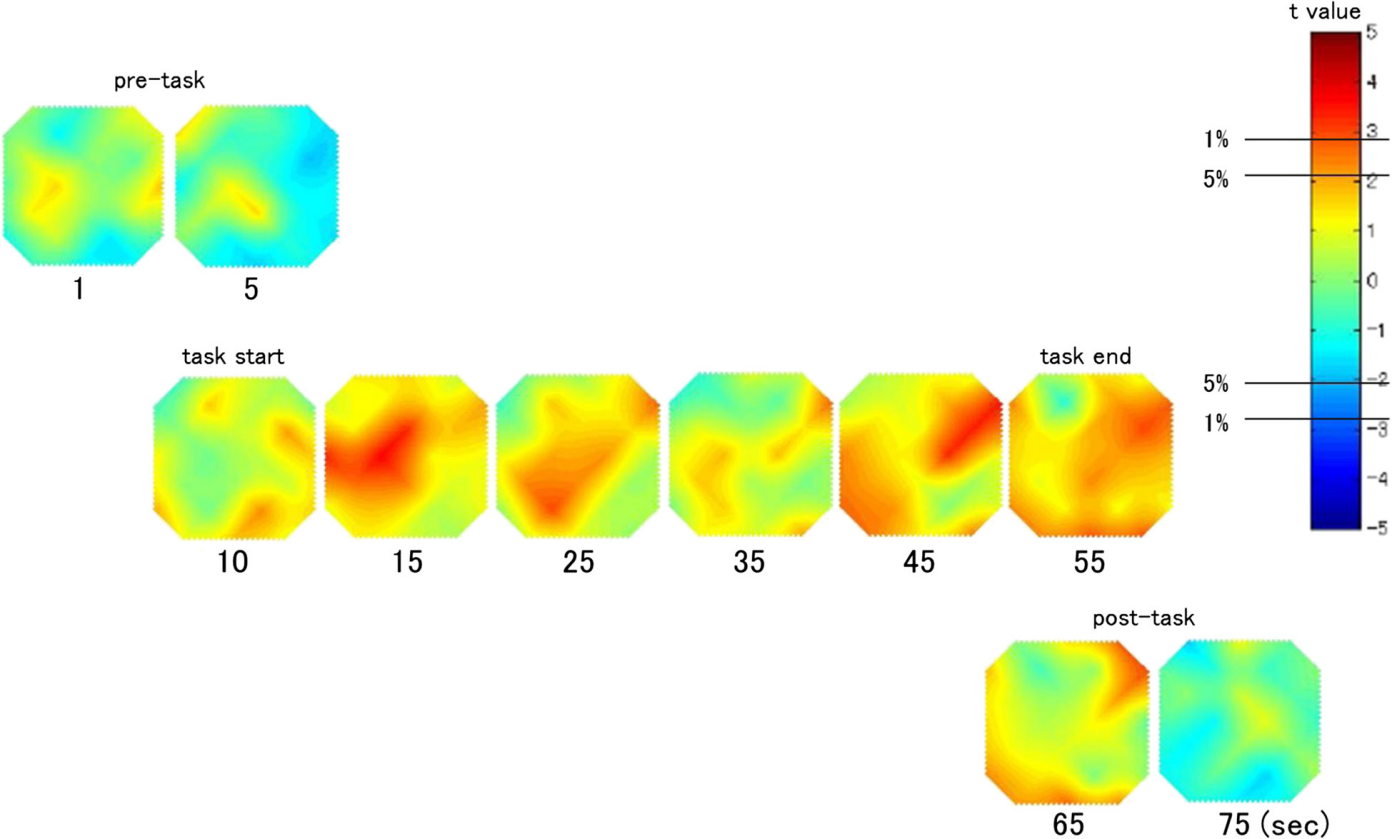
Topographic representation of *t* values corresponding to the difference in oxyhemoglobin (oxy-Hb) between the control group and the Tourette’s disorder (TD) group during the Stroop color-word task. The *t* values of oxy-Hb for the control and TD groups are presented as a topographic map along the time course of the task (from top to bottom). The red, green, and blue areas in the topographs indicate positive, zero, and negative *t* values, with ±2.8 and ±2.1 for 1% and 5% statistical significance levels, respectively.

We calculated the grand average waveforms of oxy-Hb concentration changes while both groups performed the Stroop color-word task (Figure 2). The grand waveforms of oxy-Hb concentration change increased while participants in the control group performed the task. In contrast, the grand waveforms of oxy-Hb concentration showed little change in the participants in the TD group. We found group differences in the mean oxy-Hb measurements between the pre-task and post-task periods in the 24 channels (Table 2) (FDR-corrected, all P < 0.01). Between the pre-task and post-task periods, the mean change in oxy-Hb in the TD group was significantly smaller than that in the control group in channels 7, 10, and 13. Those channels were nearly localized in the left DLPFC. Additionally, we generated a topographic representation of the t-values of oxy-Hb comparisons between the groups while the participants performed the Stroop color task (Figure 3). Overall, while performing the Stroop color task, the TD group exhibited smaller oxy-Hb changes in the prefrontal cortex, especially in the left DLPFC, compared with the control group.

### Comparison of NIRS measurements between TD patients with ADHD and those without ADHD

Our group previously reported that children with ADHD exhibit significantly smaller oxy-Hb changes in the inferior prefrontal cortex compared with healthy children [30]. This led us to investigate the grand average waveforms of oxy-Hb concentration changes between TD patients with ADHD and those without ADHD while they performed the Stroop color-word task. However, we did not find any ADHD-dependent differences in mean oxy-Hb levels in the 24 channels that we recorded (FDR-corrected, all P > 0.5).

### Comparison of NIRS measurements between TD patients with medication and those without medication

We investigated the grand average waveforms of oxy-Hb concentration changes between medicated individuals with TD and unmedicated individuals with TD while they performed the Stroop color-word task. We did not find any differences in mean oxy-Hb measurements between medicated and unmedicated TD patients in the 24 channels that we recorded (FDR-corrected, all P > 0.5).

### Correlation between NIRS measurements and participant characteristics

Because the TD patient and control groups varied considerably in terms of characteristics, we calculated Spearman’s ρ correlations for the NIRS measurements (Channels 7, 10, and 13), age, FIQ, and YGTSS scores. In the TD patient group, we found no correlations between NIRS measurements, age, FIQ, and YGTSS scores. Furthermore, in the control group, we found no correlations between NIRS measurements, age, and FIQ.

## Discussion

To the best of our knowledge, this is the first NIRS study to examine prefrontal hemodynamic response in 10 pediatric patients with TD. We found that oxy-Hb changes in the prefrontal cortex during the Stroop color-word task were significantly smaller in patients with TD compared with 10 healthy control participants.

Several previous fMRI studies of TD patients have demonstrated functional abnormalities in the frontal and/or parietal areas. fMRI has revealed that TD patients exhibit less neural activity during spontaneous tic behavior [40], and resting-state fMRI has been used to detect physiologically meaningful spontaneous low-frequency (typically 0.01–0.1 Hz) fluctuations [41]. Marsh et al. [19] compared traditional Stroop task performance in children and adults with TD with that of healthy controls during fMRI data acquisition. Among the TD patients, stronger behavioral Stroop interference effects were associated with increased DLPFC and ventrolateral PFC (VLPFC) activation. Surprisingly, in TD patients, there was a positive correlation between tic severity scores and DLPFC activation, possibly reflecting an ineffectual compensatory mechanism. As mentioned above, the results of the present study did not coincide with those of previous studies in which a similar activation task was used. However, our NIRS study included a baseline and activation task, and we referred to the relative oxy-Hb concentration as the difference in oxy-Hb concentration between these tasks. Therefore, it is possible that the participants in the TD patient group exhibited greater PFC activation, even when performing the baseline task before activation task, compared with the control participants. This might explain why, compared with the control group, the TD patient group exhibited significantly smaller differences in mean oxy-Hb concentration in channels 7, 10, and 13 between the baseline and activation tasks. Therefore, our data supported our hypothesis that an increased prefrontal hemodynamic response while performing the Stroop task would be associated with pediatric patients with TD, as has been previously identified using other imaging modalities, such as fMRI and SPECT.

The SCWC scores obtained by participants in the TD group were significantly lower than those of the control group. Low SCWC scores have been closely linked with impulsivity. Hollander et al. [42] stated that TD should be considered a mixed compulsive-impulsive disorder, along with ADHD. In addition, they stated that careful longitudinal observation could reveal a number of impulsive behaviors associated with arousal, pleasure, or gratification. Both TD and ADHD have been hypothesized to involve dysfunction in the CSTC circuits [2,9,10]. Negoro et al. [30] examined brain activation in 20 children with ADHD and 20 healthy age- and sex- matched children during the Stroop color-word task using NIRS. As in our study, they found that the SCWC scores obtained by the ADHD group were significantly lower than those obtained by the control group. Accordingly, in the present study, the lower SCWC scores obtained by the TD group may have been related to impulsivity associated with dual causes of TD and ADHD, as 5 of 10 of the participants had comorbid ADHD.

At channels 7, 10, and 13, the pediatric TD patients exhibited significantly smaller changes in oxy-Hb compared with the healthy controls. These channels are localized near the left DLPFC. Meanwhile, in the right DLPFC, pediatric TD patients did not exhibit significantly smaller changes in oxy-Hb compared with the healthy controls. Other functional and structural neuroimaging studies have described abnormalities and dysfunction in the left hemisphere. For instance, prior fMRI activation studies comparing TD children with control children reported that the left lateral frontal regions were activated in individuals with TD during a rule-switching task [43] and a Stroop task [19]. Using MRI, Peterson et al. [14] reported that children with TD had larger left DLPFC volumes relative to control children. In a SPECT study, brain abnormalities in people with TD tended to be located in the left lateral temporal lobe [44]. Additionally, reduced uptake of ethyl cysteinate dimer in the left caudate, cingulum, and DLPFC has been observed in this population. [45] These previously described lateral frontal regions are near the left DLPFC, which is implicated in our study. Previous findings regarding the left lateral frontal cortex and DLPFC activation in children with TD are consistent with our data.

There are several potential limitations of the present study. First, NIRS has certain disadvantages compared with other methodologies, namely, that NIRS enables the measurement of Hb concentration changes as relative values only, not absolute values. To address this issue, we used a version of the Stroop task wherein a baseline task was presented to the participants first (the first page). Furthermore, we measured changes in Hb concentration between the activation task and the baseline task. We also asked the participants to perform the task three times and used an average. This was done to reduce the influence of potential accidental changes and reduce participant fatigue. The grand average waveforms of changes in oxy-Hb concentration in the TD group do not show a decrease in regional cerebral flow during the activation task, although they do show differences in blood flow between the baseline and activation tasks. Second, the spatial resolution for detecting hemodynamic responses from the scalp surface using NIRS is lower than that for fMRI, SPECT, and PET. However, abnormal prefrontal hemodynamic responses in individuals with pediatric TD are certainly detectible by NIRS. Third, our sample size was relatively small. Future studies should include a larger sample size. Fourth, our study included only male participants. Future studies should also include female participants. Fifth, individuals with comorbid ADHD were not excluded from participation. However, as mentioned above, we did not find any differences in mean oxy-Hb measurements between TD patients with ADHD and those without ADHD in the 24 channels (FDR-corrected, all P > 0.5). Sixth, three participants were receiving medication during the present study. However, as mentioned above, we did not find any differences in mean oxy-Hb measurements between the medicated and unmedicated TD patients in the 24 channels (FDR-corrected, all P > 0.5).

## Conclusion

To the best of our knowledge, this is the first NIRS study to examine prefrontal hemodynamic responses while healthy participants and those with pediatric TD performed the Stroop task, as measured by NIRS. We found that changes in oxy-Hb concentration in the prefrontal cortex were significantly smaller in the TD group compared with the control group. We also found that the SCWC scores of the TD group were significantly lower than those of the control group. Our research indicates that pediatric TD patients might have prefrontal dysfunction and greater impulsivity compared with control participants. The multi-channel NIRS system appears to be a very useful tool for assessing brain function, as it enables non-invasive functional mapping of the cerebral cortex and has much shorter measurement times (about 5 min) compared with other functional brain imaging methodologies.
